# Segmental Renal Ischemia following Transplantation of Horseshoe Kidney as Separate Allografts

**DOI:** 10.1155/2013/852127

**Published:** 2013-02-17

**Authors:** J. T. Foster, P. E. Morrissey

**Affiliations:** Division of Organ Transplantation, Department of Surgery, Alpert Medical School of Brown University, 593 Eddy Street, APC 921, Providence, RI 02903, USA

## Abstract

*Introduction*. Horseshoe kidney is a congenital anomaly that presents unique challenges for the transplant surgeon. The mere presence of horseshoe kidney should not preclude consideration for transplantation. *Case Report*. A 33-year-old women suffering from end-stage renal disease underwent deceased donor renal transplant with a divided horseshoe kidney. We present a postoperative complication and the technical strategy for transplant salvage. The patient currently has excellent graft function. *Discussion*. Horseshoe kidneys do present challenges for successful transplantation. Though case reports of successful transplantation are increasing, we present a technical complication and successful transplant salvage strategy. Technical descriptions in the literature of successful back-table preparation strategies should help more transplant surgeons to begin to utilize this resource. *Conclusion*. This study concludes that horseshoe kidneys can be successfully used for transplantation and provides a technical strategy to salvage the transplant after a unique complication associated with these donor kidneys.

## 1. Introduction

 Horseshoe kidney is a congenital anomaly found in approximately 1 in 400 persons. With 6,000 to 8,000 deceased donors per year in the United States, utilization of horseshoe kidneys for renal transplantation would afford more organs for transplant recipients. The first case reports of transplanted horseshoe kidneys were described in 1975 by Nelson and Palmer [1]. A recent review found only 55 reported cases of transplanted horseshoe kidney in the literature [2]. Of these cases, 15 were transplanted en bloc. Two were split horseshoe kidneys from living donors, and the remaining 38 were split from cadaveric donors and then transplanted.

 Several factors must be considered when deciding to utilize a horseshoe kidney for transplantation including renal size (a surrogate for nephron mass), renal vascular anomalies, and urologic anatomy. Only one-third of horseshoe kidneys have normal renal arterial supply [2]. As the kidney is supplied by functional end arteries, failure to preserve inflow to the kidney will result in ischemia. We report a unique urological complication encountered in one of the two renal transplant recipients related to aberrant blood supply in a horseshoe kidney.

## 2. Case Report

### 2.1. Donor Anatomy and Surgery

**Figure 1:**
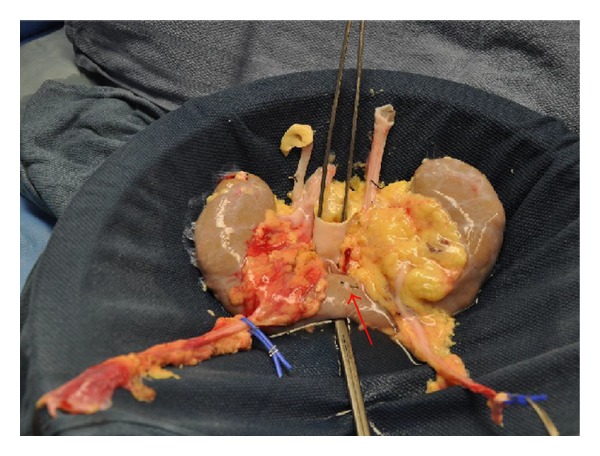
Horseshoe kidney. The red arrow points to the accessory artery feeding the bridge of the horseshoe kidney. The indicated artery was ligated during back-table preparation of the kidneys.

**Figure 2:**
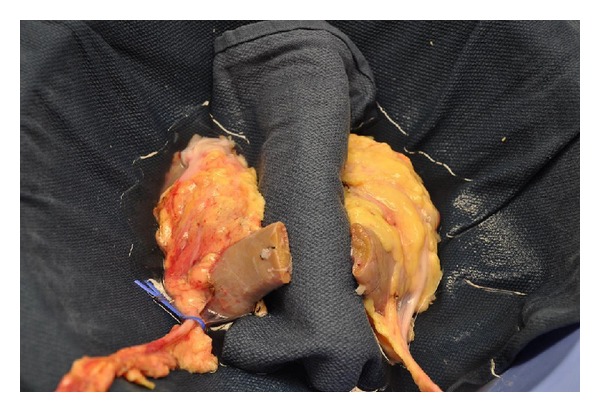
Divided kidneys. The cut ends were oversewn with pledgeted no. 1 Vicryl sutures in preparation for transplantation of the two kidneys into recipient patients.

 The kidneys from a 55-year-old donor following a cerebrovascular accident and brain death were accepted for transplantation. The donor had a congenital horseshoe kidney and normal creatinine (0.8 mg/dL). Each half of the horseshoe kidney was of normal size and each kidney was drained by a single ureter. A single renal artery and renal vein supplied each allograft in the hilum. An additional artery originated from the donor iliac artery and supplied the joining bridge (isthmus) at the lower poles. This artery measured 3 mm in diameter and was transected during organ recovery. The fusion of the lower poles measured 8 mm in thickness and 3 cm in diameter (Figure 1). The accessory artery was ligated. The kidney was divided at the parenchymal bridge and oversewn with pledgeted no. 1 Vicryl sutures on the back table (Figure 2). Two recipients were identified and each received one half of the recovered horseshoe kidney. At the time of transplantation, each kidney demonstrated suboptimal perfusion at the divided edge.

### 2.2. Recipient Surgery

 The recipient was a 33-year-old woman with end-stage renal disease due to IgA nephropathy who had been on hemodialysis for approximately 2.5 years at time of transplantation. The single kidney was prepared as mentioned above. The cold ischemia time of the kidney was 22 hours and the anastomosis time was 26 minutes. The operation was performed without complication. During the first postoperative day, the patient's urine output was 600 milliliters and the creatinine rose, but on subsequent days the creatinine steadily declined, consistent with slow allograft function. Dialysis was not required. A rise in serum creatinine on the fifth postoperative day prompted a renal ultrasound, which demonstrated normal renal blood flow and no fluid collections or obstruction. On the seventh postoperative day, the patient's creatinine again increased (from 3.4 mg/dL to 3.9 mg/dL). A MAG3 nuclear study demonstrated a urine leak. A Foley catheter was placed for bladder decompression, and a retrograde cystogram showed no extravasation of contrast. The patient's creatinine steadily declined and she was discharged home with a Foley catheter. On postoperative day 17, the patient was seen in the outpatient clinic. The serum creatinine was 1.8 mg/dL, and the wound was healing well without signs of drainage. The Foley catheter was removed.

**Figure 3:**
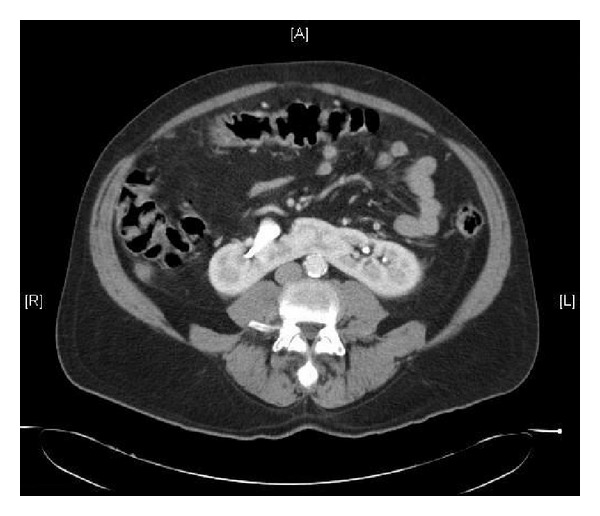
Donor CT scan. This image from the prerecovery donor CT scan shows the bridge of the horseshoe kidney and the inferior most portion of the collecting system noted on CT scan.

 Four days later, the patient presented to our emergency department with 3-day history of abdominal pain and fevers. Her creatinine had increased to 2.5 mg/dL. A CT scan of the abdomen and pelvis showed fluid collections around the transplanted kidney. She was taken to the operation room for exploration of the renal transplant. The lower pole of the transplanted kidney including the retained segment of the isthmus was ischemic. This comprised less than 5% of the total renal volume. The pledgets placed at time of transplantation were disrupted from the lower pole and the Vicryl sutures were absorbed. Intraoperatively, the patient was administered indigo carmine intravenously, which was excreted from the transected end of the lower pole of the kidney. There was no evidence of urine leak from the renal pelvis, ureter, or bladder. The ischemic renal tissue was excised and a 7-8 mm defect corresponding to the collecting system was identified. This area corresponded to the most inferior extent of the collecting system noted on the prerecovery donor CT scan (Figure 3). The debrided edge was cauterized and the identified defect was closed in 3 layers with a 2-0 chromic suture in the collecting system and several 2-0 PDS U-stitches through the renal parenchymal tissue and a more proximal row of no. 1 PDS sutures to reinforce the closure. The patient was discharged home five days after reexploration with a Foley catheter, which was removed a few days later in the outpatient clinic. She presently enjoys excellent renal graft function with a serum creatinine level of 1.62 mg/dL. The paired kidney, transplanted into a 55-year-old, African-American patient with end-stage renal disease due to diabetes, achieved a similar nadir of 1.6 mg/dL lacking any urological sequelae.

## 3. Discussion

 The urologic literature reveals an increasing number of reports of successfully transplanted horseshoe kidneys, but little information is available on the preparation of such kidneys. Uzzo et al. proposed an algorithm for the evaluation and utilization of horseshoe kidneys for cadaveric transplantation [3]. Renovascular anatomy, ureteral anatomy, or ureter length variations should not preclude the use for transplantation. Degree of fusion is addressed and kidneys with a thick isthmus are recommended to be transplanted en bloc; however, the determination of thick versus thin isthmus is left to the surgeon's discretion. Another report described the strategies for the closure of the divided renal parenchyma including using a GIA stapler device or oversewing with absorbable suture [4]. In most cases, the isthmus bridge consists of only a fibrous band of renal parenchyma. Rarely does the urinary collecting system cross the bridging isthmus. In the cases of thick isthmus, one study suggests contrast evaluation of the collecting system and transplantation of the horseshoe kidney en bloc if the collecting system crosses the isthmus [5]. A potential strategy in our case would have been to reimplant the accessory artery. We elected not to do so as the artery entered the midportion of the isthmus, and preserving the blood supply would have complicated dividing the kidneys. Another option would have been to inject the accessory artery to the isthmus with methylene blue to define the extent of parenchymal blood supply by this vessel. Resection of the isthmus with a more proximal oversewing of the kidney at the initial operation may have prevented the ensuing necrosis and urine leak.

 Horseshoe kidneys are an underutilized resource that should not be rejected for potential deceased donor transplant unless careful examination of the kidneys reveals prohibitively complex vascular or ureteral anatomy. Careful evaluation of the bridging tissues is essential for successful division or appropriate decision to transplant a horseshoe kidney en bloc. Once a horseshoe kidney is divided, the exposed parenchyma must be closed to assure hemostasis and prevent urinary fistulas. The manner and location in which the isthmus is divided should be based on size, proximity to the collecting system, and, as was critical in this case, blood supply. This paper demonstrates that satisfactory outcomes can be achieved by transplanting two patients with a divided horseshoe kidney. We offer a caveat regarding the management of vascular abnormalities at the retained isthmus that resulted in an unusual mechanism of urinary leak.

## References

[1] Nelson RP, Palmer JM (1975). Use of horseshoe kidney in renal transplantation: technical aspects. *Urology*.

[2] Pontinen T, Khanmoradi K, Kumar A (2010). Horseshoe kidneys: an underutilized resource in kidney transplant. *Experimental and Clinical Transplantation*.

[3] Uzzo RG, Hsu THS, Goldfarb DA, Taylor RJ, Novick AC, Gill IS (2001). Strategies for transplantation of cadaveric kidneys with congenital fusion anomalies. *Journal of Urology*.

[4] Tan HP, Samaniego MD, Montgomery RA (2001). Donor horseshoe kidneys for transplantation. *Transplantation*.

[5] Stroosma OB, Schurink GWH, Smits JMA, Kootstra G (2001). Transplanting horseshoe kidneys: a worldwide survey. *Journal of Urology*.

